# Esophageal Liposarcoma: A rare tumor

**DOI:** 10.12669/pjms.40.2(ICON).8989

**Published:** 2024-01

**Authors:** Ameet Kumar, M. Saqib Qamar Ishaqi, Mehak Ali Baig, Rafeah Khan

**Affiliations:** 1 Ameet Kumar, MBBS, Resident Radiology, Indus Hospital and Health Network, Karachi, Pakistan; 2 Saqib Qamar Ishaqi, MBBS, FCPS, EDIR, Consultant Radiologist, Indus Hospital and Health Network, Karachi, Pakistan; 3 Mehak Ali Baig, MBBS, Resident Radiology, Indus Hospital and Health Network, Karachi, Pakistan; 4 Rafeah Khan, MBBS, FCPS, EDIR, Head of Department, Consultant Radiologist, Indus Hospital and Health Network, Karachi, Pakistan

**Keywords:** Esophageal liposarcoma, Lipomatous tumors

## Abstract

Esophageal liposarcoma is a rare type of tumor. This case report documents the presentation, diagnosis, and management of a 74-year-old male with a medical history including diabetes mellitus, asthma, and hypertension. The patient’s primary complaint was dysphagia, accompanied by regurgitation and substantial weight loss over a six-month period. Diagnostic evaluation revealed a sizable esophageal liposarcoma, which was successfully resected through surgery. Follow-up assessments demonstrated the absence of residual mass. Esophageal liposarcomas, though rare, should be considered in patients presenting with dysphagia or chest discomfort. Surgical resection is the mainstay of treatment, with the recommendation for extended postoperative surveillance given the limited available data regarding long-term prognosis.

## INTRODUCTION

Primary esophageal liposarcoma is an extremely rare tumor that arises from adipocytes cells in esophagus and account for less than 1% of all esophageal tumors. First report was published in 1983^1^ with only 63 cases reported in literature since then.^2^

## CASE REPORT

Seventy four years old male, known case of diabetes mellitus, asthma and hypertension presented in the hospital with complaints of dysphagia for last six months. He had frequent problems with food being stuck, along with regurgitation for last one month and was only able to tolerate a few sips of water. This was also associated low-grade fever and weight loss for the last two months.

During the hospital admission, endoscopy was performed which showed a smooth bulge in the esophagus from 15cm to 38cm occupying 3/4^th^ of its lumen. Ulcerated mucosa was seen in the distal part of the esophagus. CT scan chest abdomen with I/V contrast was performed which showed a huge pedunculated, lobulated mass lesion almost completely occupying the esophageal lumen with resulting significant luminal narrowing. There was evidence of patchy intralesional fat components with an average Hounsfield unit within the lesion was (-100). Overall findings were favoring esophageal liposarcoma. Laparotomy and posterolateral thoracotomy were performed which showed a pedunculated papillomatous growth measuring 25cm in the esophagus with its stalk in the cervical portion of esophagus. Gastro-esophageal junction was clear as per surgical findings.

Complete surgical resection of the growth was done and sample was sent for histopathology. As per histopathology report biopsy shows a giant polyp covered by stratified squamous epithelium. Underlying stroma shows well differentiated lipomatous areas admixed with fibrous component showing scattered large atypical stromal cells. Mitoses and areas of necrosis were present. Differential diagnoses included atypical lipomatous tumor / well differentiated liposarcoma and giant fibrovascular polyp. Based on the presence of numerous bizarre atypical stromal cells, necrosis and mitoses, a possibility of well differentiated liposarcoma was favored. Thus, histopathology confirmed the diagnosis of well differentiated esophageal liposarcoma. On post-surgical follow up barium swallow and meal were performed which showed contrast opacification of esophagus with normal passage up to stomach. No luminal narrowing or filling defect was seen to suggest residual mass lesion.

**Fig.1:**
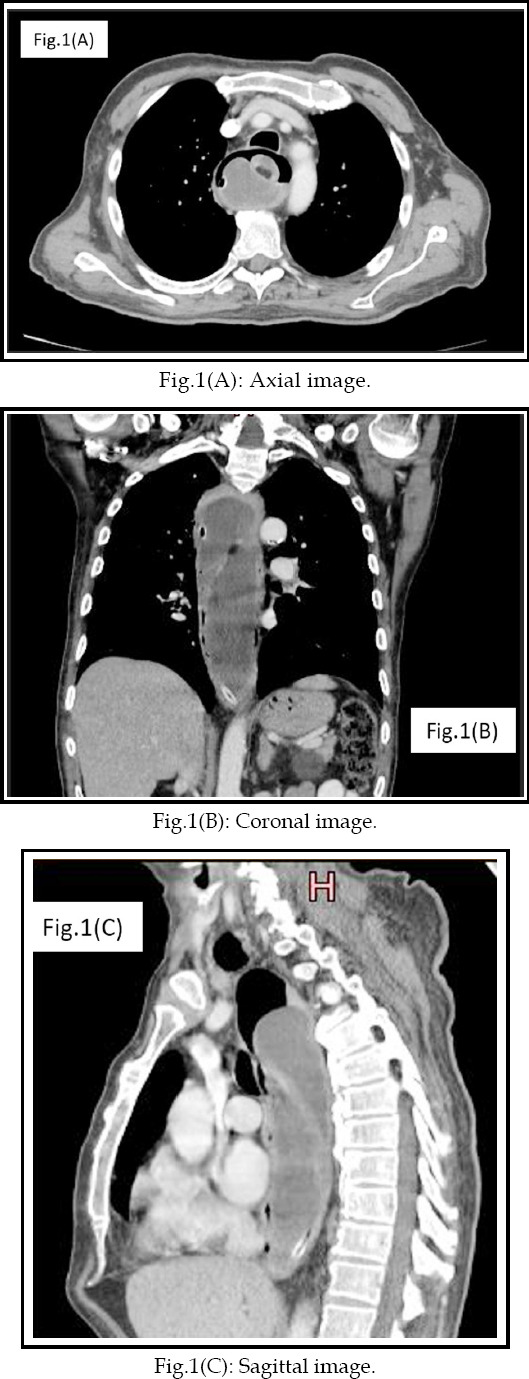
Findings of the contrast-enhanced computed tomography. Huge lobulated mass lesion in esophagus almost completely occupying its lumen. There is evidence of patchy intralesional fatty components. NG tube is also partially visualized.

**Fig.2:**
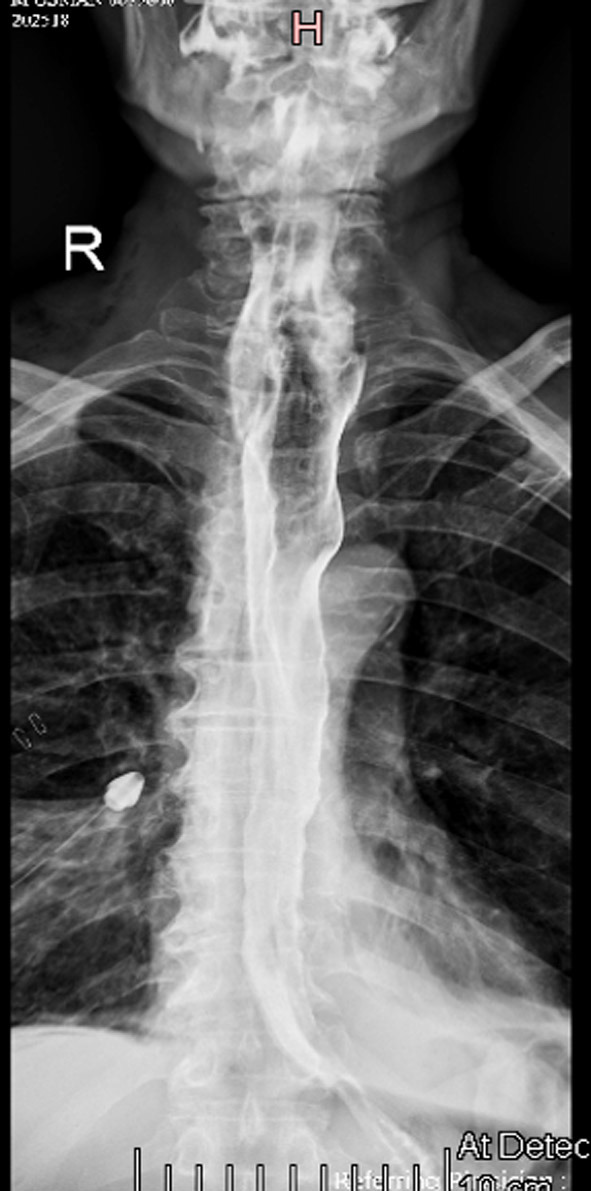
POST SURGERY. Spot image of barium swallow. This shows normal contrast opacification of esophagus without evidence of filling defect or luminal narrowing. No contrast extravasation is noted. Tip of post-surgical drain is noted on the right. Few surgical staples are also seen close to drain.

## DISCUSSION

Lipomatous tumors of the gastrointestinal tract are usually found in the distal ileum and large bowel. Liposarcoma rarely develop in the aero-digestive tract. The mucosal and submucosal esophageal layers rarely give rise to primary esophageal liposarcoma. Based on data of few available cases esophageal liposarcoma tend to occur predominantly in males (72%) at an average age of 58.4 years and reaching an average size of 13cm (range 4-23cm). These are usually slowly growing tumors with lesion usually grows until it reaches in considerable size.^3^

Predominant clinical symptoms are progressive dysphagia, anemia, foreign body sensation and regurgitation. Sometimes symptoms are not characteristic like shortness of breath, cough and chest discomfort making it difficult to diagnose.^3^,^4^ Radiographs are nonspecific and show widening of mediastinum and lung pathologic abnormalities such as pleural or parenchymal metastasis if present. CT scan remains the most important diagnostic modality. Imaging appearances include large heterogenous predominantly fat-density lesion mixed with varying amounts of soft tissues. MRI shows abnormal signal intensity lesion with fat signals on T1 weighted images and shows suppression on fat-suppressed images.^5^ In and out-of-phase sequences are also helpful in diagnosis.

The treatment of choice is surgical resection. As these tumors are usually pedunculated and well differentiated, they can be safely resected locally with good 5-year survival rate of 75-100%. However, exact prognosis of liposarcoma after radical surgery remains unknown as only limited numbers of cases are published in literature.^6^ As per available medical records only 2/15 cases have been reported for recurrence after radical surgery.^7^ Due to this adjuvant therapy is not suggested in these patients. Surgery with long term follow up for surveillance is usually recommended to see any disease can recurrence.^7^,^8^

## CONCLUSION

Esophageal liposarcoma is a rare tumor that should be considered in the differential diagnosis of patients with dysphagia or chest pain. CT and MRI are useful imaging modalities for the diagnosis of esophageal liposarcoma. Surgical resection is the treatment of choice for esophageal liposarcoma. Due to limited published cases insufficient data is available regarding post-surgical prognosis. But long term follow up is usually recommended to see any disease recurrence.

### Authors’ Contribution:

**AK:** Manuscript writing, literature search, patient follow up and are responsible for accuracy and integrity of the report

**MSQI & RK:** Radiological diagnosis, interpretations of radiological images

**MAB:** Literature review.
